# Recovery of Synthetic Zika Virus Based on Rio-U1 Isolate Using a Genetically Stable Two Plasmid System and cDNA Amplification

**DOI:** 10.3389/fmicb.2021.639655

**Published:** 2021-02-24

**Authors:** Iasmim Silva de Mello, Déberli Ruiz Fernandes, Nathália Dias Furtado, Alexandre Araújo Cunha dos Santos, Marta Pereira dos Santos, Ieda Pereira Ribeiro, Lidiane Menezes Souza Raphael, Mônica da Silva Nogueira, Stephanie Oliveira Diaz da Cruz, Adalgiza da Silva Rocha, Pedro Paulo de Abreu Manso, Marcelo Pelajo-Machado, Myrna Cristina Bonaldo

**Affiliations:** ^1^Laboratório de Biologia Molecular de Flavivírus, Instituto Oswaldo Cruz, Fundação Oswaldo Cruz, Rio de Janeiro, Brazil; ^2^Centro de Experimentação Animal, Instituto Oswaldo Cruz – FIOCRUZ, Rio de Janeiro, Brazil; ^3^Central Analítica, Unidade de Apoio ao Diagnóstico do COVID-19 - UNADIG-RJ, Vice-Presidência de Produção e Inovação em Saúde – FIOCRUZ, Rio de Janeiro, Brazil; ^4^Laboratório de Patologia, Instituto Oswaldo Cruz, Fundação Oswaldo Cruz, Rio de Janeiro, Brazil

**Keywords:** Zika virus, infectious clone, two-plasmid system, cDNA amplification, cell infection, AG129 mouse infection

## Abstract

In 2016, the world experienced the unprecedented Zika epidemic. The ZIKV emerged as a major human pathogen due to its association with the impairment of perinatal development and Guillain–Barré syndrome. The occurrence of these severe cases of Zika points to the significance of studies for understanding the molecular determinants of flavivirus pathogenesis. Reverse genetics is a powerful method for studying the replication and determinants of pathogenesis, virulence, and viral attenuation of flaviviruses, facilitating the design of vaccines and therapeutics. However, the main hurdle in the development of infectious clones is the instability of full-length cDNA in *Escherichia coli*. Here, we described the development of a genetically stable and efficient infectious clone based on the ZIKV Rio-U1 isolated in the 2016 epidemic in Brazil. The employed strategy consisted of cloning the viral cDNA genome into two stable plasmid subclones and obtaining a high-quality cDNA template with increment in DNA mass for *in vitro* transcription by PCR amplification. The strategy for developing a ZIKV infectious cDNA clone designed in this study was successful, yielding a replicative and efficient clone-derived virus with high similarities with its parental virus, Rio-U1, by comparison of the proliferation capacity in mammal and insect cells. The infection of AG129 immunocompromised mice caused identical mortality rates, with similar disease progression and morbidity in the animals infected with the parental and the cDNA-derived virus. Histopathological analyses of mouse brains infected with the parental and the cDNA-derived viruses revealed a similar pathogenesis degree. We observed meningoencephalitis, cellular pyknosis, and neutrophilic invasion adjacent to the choroid plexus and perivascular cuffs with the presence of neutrophils. The developed infectious clone will be a tool for genetic and functional studies *in vitro* and *in vivo* to understand viral infection and pathogenesis better.

## Introduction

Flaviviruses are the most critical arthropod-borne viruses and are of worldwide clinical concern (Chong et al., 2019; Pierson and Diamond, 2020). Zika virus (ZIKV, family *Flaviviridae*, genus *Flavivirus*) was associated with a massive outbreak of a febrile illness spread throughout the Americas (Kindhauser et al., 2016). It became a global public concern due to the correlation of the ZIKV epidemic with severe diseases, such as congenital syndrome in neonates and Guillain–Barré syndrome in adults (Sips et al., 2012; Costello et al., 2016; Rasmussen et al., 2016; Pierson and Diamond, 2018).

The ZIKV is mainly transmitted by *Aedes* sp. mosquitoes (Musso and Gubler, 2016). Furthermore, non-vector transmission through sexual contact or vertical routes from infected mothers have also been reported (Musso et al., 2015; Cao et al., 2017), posing new challenges for controlling outbreaks caused by this virus. To date, there is no effective licensed vaccine or antiviral treatment against ZIKV infection (Shan et al., 2018; Bernatchez et al., 2019).

The viral genome is a positive single-stranded RNA approximately 11 kb in length, encoding 3,423 amino acids. There are two untranslated regions at each genome end (5′ and 3′ UTRs) and a single open reading frame. The viral RNA is translated into a precursor polyprotein, which is cleaved co- and post-translationally into three structural proteins (C, prM/M, and E) and seven non-structural proteins (NS1, NS2A, NS2B, NS3, NS4A, NS4B, and NS5)(Kuno and Chang, 2007; Musso and Gubler, 2016).

The availability of genetic tools for studying viruses, such as infectious cDNA clones, allows for assessing genetic factors related to viral pathogenesis, immune response, and viral evolution. The use of infectious clones allows the development of effective vaccines and antiviral therapeutics (Weger-Lucarelli et al., 2017b; Avila-Perez et al., 2020).

Reverse genetics is a powerful method for studying the viral replication of RNA viruses and has been widely used for positive-stranded RNA viruses, such as Flaviviruses (Ruggli and Rice, 1999; Stobart and Moore, 2014). However, the instability of full-length flavivirus cDNA in *Escherichia coli* has been a significant hurdle in attempting to construct infectious clones due to active prokaryotic cryptic promoters present in their genomes (Pu et al., 2011). Several strategies have been developed to overcome this problem, including splitting the genome into different plasmids (Rice et al., 1989) and using low-copy number vectors (Shan et al., 2016; Shan et al., 2017a).

Based on our previous experience with the vaccine YFV-17D infectious clone (Bonaldo et al., 2002, 2007; Nogueira et al., 2011; de Santana et al., 2014), we designed a similar strategy splitting the genome into two stable plasmid subclones, with subsequent assembly and viral regeneration in Vero cells. However, to mitigate toxicity and reach recombinant plasmid genetic stability, we constructed them utilizing a low-copy number vector (pCC1). In a further step, we performed the polymerase chain reaction (PCR) to increase the amount of viral cDNA and recover a high-quality template for *in vitro* transcription. The strategy for developing a ZIKV infectious cDNA clone designed in this study was successful, yielding a replicative and efficient clone-derived virus with high similarities with its parental virus, Rio-U1. These results expand the potential use of reverse genetic systems and open the possibility of employing similar approaches for other flaviviruses, which could be a suitable platform for studying viral determinants of pathogenesis, virulence, and viral attenuation, facilitating the design of vaccines and therapeutics.

## Materials and Methods

### Cells

The African green monkey kidney (Vero) cell line (ATCC- CCL81) was grown at 37°C, under an atmosphere containing 5% CO_2_, in Earle’s 199 medium supplemented with 5% fetal bovine serum (FBS) and 40 μg/ml of gentamicin. *Aedes albopictus* cell line (C6/36) was generously provided by Anna-Bella Failloux (Institut Pasteur, France) and was grown at 28°C, in Leibovitz’s L-15 medium (Gibco), supplemented with 5% FBS and 10% Tryptose broth.

### ZIKV Isolate and Synthetic cDNA Fragments

The ZIKV strain Rio-U1 (GenBank accession number: KU926309) was isolated in Rio de Janeiro, Brazil, in 2016 from a human patient’s urine sample (Bonaldo et al., 2016). The ZIKV cDNA was synthesized in four fragments (GenScript). Fragments Z1, Z2, and Z4 were cloned into a high-copy plasmid pUC57 while fragment Z3 was cloned into a derivative of the low-copy plasmid pCC1-Fos (Epicenter). pCC1 is based on a previously developed technology (Carson et al., 1973). It is a single copy plasmid usually under control of the *E. coli* F factor single-copy origin of replication. But in the presence of an inductor (L-arabinose) the initiation of replication from high-copy oriV origin occurs, which requires the trfA gene product supplied by the *E. coli* cells. All fragments encompassed the entire genome of the ZIKV Rio-U1, with overlapping regions at their ends. To ensure a seamless assembly of the full-length cDNA, we engineered silent mutations to introduce restriction cleavage sites at both ends of each synthetic fragment.

### Infectious Clone Construction

**FIGURE 1:**
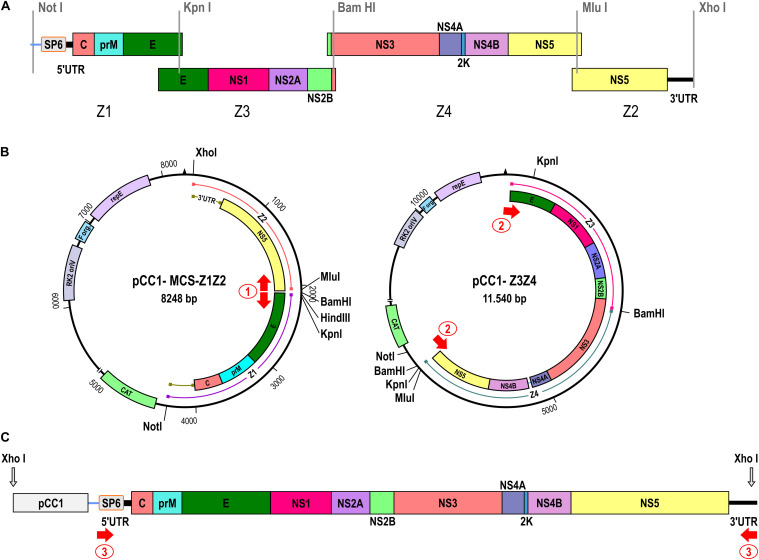
Construction of the ZIKV Rio-U1 infectious clone. **(A)** Four cDNA fragments were synthesized spanning the entire ZIKV genome as depicted in the diagram. The promoter for the SP6 RNA polymerase was fused to the 5′ end of the ZIKV cDNA (Z1). Restriction sites used for the assembly of the fragments generating the infectious clone are indicated. **(B)** The two-plasmid system of the ZIKV infectious clone is composed by the pCC1-MCS-Z1Z2 and pCC1-Z3Z4 plasmids carrying the 5′ and 3′ ends and the central part of the ZIKV genome, respectively. Single restriction-enzyme sites utilized for the infectious clone assembly are indicated. The red arrows show the regions of primer sets 1 and 2 annealing for ZIKV cDNA amplification. **(C)** Schematic representation of the complete ZIKV template. The red arrow numbered 3 represents the region of primer annealing utilized to amplify and increase the template mass.

To construct the ZIKV infectious clone utilizing the two-plasmid system approach, the fused fragments bearing either the extremities or the center of the viral genome were cloned into pCC1 plasmid by modification of pCC1-Z3. *E. coli* strain TransforMax EPI300 (Epicenter) was used to propagate the plasmids. The two plasmids constructed were the pCC1-Z3Z4 encoding the central part of the ZIKV genome (genome regions: Z3, from 2,002 to 4,776 and, Z4, from 4,777 to 8,843), and pCC1-MCS-Z1Z2 (genome regions: Z1, from 1 to 2,001 and, Z2, from 8,844 to 10,807) carrying the 5′ and 3′ends of the viral genome (Table 1). To assemble these plasmids, we digested individual cDNA plasmids with specific restriction endonucleases, excised DNA fragment from 0.8% agarose gel/TAE after electrophoresis, followed by purification using QIAquick Gel Extraction Kit (Qiagen). Fragment Z4 was excised from pUC57 plasmid by cleaving with the restriction enzymes *Kpn*I and *Bam*HI, cloned into the likewise *Kpn*I/*Bam*HI digested pCC1-Z3 plasmid, generating the pCC1-Z3Z4 plasmid. To assemble the pCC1-MCS-Z1Z2 plasmid, pCC1-Z3 plasmid was treated with the restriction enzymes *Not*I and *Nsi*I to replace the fragment Z3 with a polylinker sequence (Supplementary Figure 1), which has the complementary restriction sites at the ends. This replacement originated a new vector named pCC1-MCS (pCC1 + multiple cloning site). Then, Z1 and Z2 were excised from pUC57 plasmid with *Not*I/*Kpn*I and *Mlu*I/*Xho*I cleavages, respectively, cloned into pCC1-MCS, and also digested with *Not*I/*Kpn*I and *Mlu*I/*Xho*I one after the other (Figure 1). The sequence of each constructed plasmid was validated by DNA sequencing before it was used in subsequent steps. The Z3Z4 fragment and the entire pCC1-MCS-Z1Z2 were amplified by PCR using Phusion High-Fidelity DNA polymerase (Thermo Fisher Scientific) and primer pairs 1 and 2 (Supplementary Table 2 and Supplementary Figure 2). Both amplicons obtained by PCR were digested with *Kpn*I and *Mlu*I restriction enzymes. After cleavage, the fragments were gel purified, as described above. The isolated fragments were ligated, yielding the full-length ZIKV cDNA in pCC1 plasmid, followed by linearization using restriction enzyme *Xho*I, generating the 3′ end of the ZIKV intermediate template (Supplementary Figure 2). The complete ZIKV cDNA template was amplified using PrimeStar GXL DNA polymerase (Takara) and primer pair 3, allowing to increase the template mass for the transcription to obtain the viral RNA, and also adding the nucleotide T at the 3′ end (previously removed by the *Xho*I cleavage) (Supplementary Figure 2).

**TABLE 1:** ZIKV cDNA fragments spanning the whole viral genome used in viral template assembly.

cDNA fragment (genomic position)	Initial cloning vector	pCC1-MCS-Z1Z2 (genomic position)	pCC1-Z3Z4 (genomic position)	Template (genomic position)	Cloning sites
Z1 (1–2,047)	pUC57	1–2,001	–	1–2,001	*Not*I – *Kpn*I
Z3 (1,653–4,793)	pCC1-4K	–	2,002–4,776	2,002–4,776	*Kpn*I – *Bam*HI
Z4 (4,581–8,855)	pUC57	–	4,777–8,843	4,777–8,843	*Bam*HI – *Mlu*I
Z2 (8,844–10,807)	pUC57	8,844–10,807	–	8,844–10,807	*Mlu*I – *Xho*I

### *In vitro* Transcription and Cell Transfection

The final amplicon containing the full-length ZIKV cDNA was purified by precipitation with ethanol and 10% ammonium acetate (3M) and resuspended in 8 μl 10 mM Tris-HCl, pH 8.5. The mMESSAGE mMACHINE SP6 transcription kit (Ambion) was used to transcribe the viral cDNA template in a 20 μl reaction. The reaction mixture was incubated at 37°C for 2 h, followed by the addition of 2 U TURBO DNAse (Ambion). The synthetic RNA was quantified using Qubit Fluorometer (Thermo Fisher Scientific). Subsequently, 2 μg of transcribed RNA was electroporated into 1 × 10^6^ Vero cells in 4 mm cuvettes with the GenePulserXcell (Bio-Rad) at settings of 200 V and 850 μF, pulsing one time. After electroporation, the transfected cells were seeded in a T-25 flask with 12 ml of Earle’s 199 medium (Gibco) supplemented with 5% fetal bovine serum (FBS) (Gibco), 5% sodium bicarbonate and 40 mg/ml gentamicin, followed by incubation at 37°C and 5% CO_2_. The cells were daily monitored for cytopathic effect (CPE). The viral recovery was confirmed by RT-PCR utilizing the primer set ZK2F-ZK2R (genomic region from 1,732 to 3,385; Supplementary Table 2). The distinction between Rio-U1 and the infectious clone was performed by treatment with restriction enzyme *Kpn*I. The supernatant was harvested at 5 days post-transfection, clarified by centrifugation at 700 g, and stored in aliquots at −80°C. The integrity of the genome of the ZIKV IC.RioU1 was confirmed by nucleotide sequencing.

### Nucleotide Sequencing Analysis

Viral RNA was extracted from culture supernatants with the QIAamp Viral RNA Mini Kit (Qiagen) according to the manufacturer’s recommendations and stored at −80°C until use. The nucleotide sequencing of the full-length ZIKV genome was performed from a set of 8 overlapping amplicons obtained by RT-PCR. The primers designed for the RT-PCR and viral genome sequencing are listed in Supplementary Table 2. The cDNA was obtained by reverse transcription using the Superscript IV First-Strand Synthesis System (Invitrogen), followed by the amplification with GoTaq Green Master Mix (Promega) according to the manufacturer’s instructions. The plasmids generated in this work were also sequenced using the same primer set. The nucleotide sequencing reactions were performed by ABI BigDye terminator V3.1 Ready Reaction Cycle Sequencing Mixture (Applied Biosystems). The nucleotide sequences were determined by capillary electrophoresis at the sequencing facility of Fiocruz-RJ (RPT01A -Sequenciamento de DNA- RJ). The sequences were assembled using the SeqMan Pro version 8.1.5 (DNASTAR, Inc.).

### Indirect Immunofluorescence Assay

The expression of the ZIKV envelope (E) protein was analyzed by indirect immunofluorescence assay (IFA). Vero cells were seeded at a density of 30,000 cells/cm^2^ in 8-well glass slides (Lab-Tek Chamber Slide System -Nunc) and incubated at 37°C with 5% CO_2_ overnight. Cells were infected with 100 μl of the recombinant or parental virus at MOI of 0.02. After 1 h at 37°C with 5% CO_2_, the inoculum was removed, and 300 μl of supplemented Earle’s 199 medium was added to the cells. After 72 h of incubation, cells were washed with PBS and fixed with 4% paraformaldehyde solution for 10 min at room temperature. After three washing steps with PBS, the cells were permeabilized with 0.5% Triton-X100 in PBS for 15 min and then incubated for 10 min with blocking buffer (1% BSA in PBS) at room temperature. The cells were incubated with the primary antibody 4G2 (3 mg/ml- Biomanguinhos) diluted 1: 1000 in blocking solution for 1 h at room temperature. After washing with PBS, the secondary antibody (IgG against mouse coupled to Alexa Fluor 546 - Molecular Probes) was added at the 1: 500 dilution in blocking buffer. Finally, the slides were mounted in a medium with SlowFade Antifade DAPI (Molecular Probes). Fluorescence analyses were performed initially on the IX51 inverted microscope (Olympus), and images were acquired through the DP Controller program and viewed through the DPManager program (DP-BSW v3.1 - Olympus).

### Plaque Morphology Assays

The plaque phenotypes of infectious clone and parental ZIKV were compared by plaque size and immunofocus assays. In the plaque size assay, Vero cells were seeded at a density of 50,000 cells/cm^2^ in six-well plates. After 24 h, the medium was replaced with 200 μl of 10-fold serial dilutions of each virus for 1 h at 37°C with 5% CO_2_. Viral inocula were removed and replaced with 3 ml of fresh supplemented Earle’s 199 medium containing 0.5% of agarose (Invitrogen). After 5 days of incubation at 37°C with 5% CO_2_, cells were fixed with 10% formaldehyde, washed, and stained with 0.4% violet crystal to visualize plaques. Images of the plates were acquired, and plaque sizes were measured using ImageJ software. The plaque areas’ means and standard errors were calculated. Differences in plaque areas’ means were analyzed using the unpaired *t*-test (Two-tailed).

We performed the immunofocus assay as described elsewhere (Magnani et al., 2017). Vero cells seeded at a density of 30,000 cells/well in 96-well plates were infected with 10-fold serial dilutions of the viruses in supplemented Earle’s 199 medium. Inocula were removed after viral adsorption. Then, cell monolayers were overlaid with 150 μl of the same viral growth medium containing 1% CMC (carboxymethyl cellulose; Gibco). After 2 days, cells were fixed with BD Cytofix/Cytoperm solution (BD Biosciences) at room temperature for 30 min, washed twice with PBS, and treated with CytoPerm Wash (BD Biosciences) for 5 min, followed by 1 h incubation with the primary antibody 4G2. Plates were washed three times, followed by an hour-long incubation with a secondary antibody goat anti-mouse IgG conjugated to peroxidase (KPL). Detection proceeded with the addition of True Blue Peroxidase Substrate (KPL), following the manufacturer’s instructions. The number of foci was analyzed with a CTL Immunospot instrument. The areas of foci were measured, and the mean and standard error of the focus areas were calculated.

### Viral Titration by Plaque Assay

Virus titers were determined by plaque assay. Vero cells were seeded at a density of 50,000 cells/cm^2^ in 24-well plates 24 h before inoculation. In brief, 100 μl of 10-fold serial dilutions of the virus samples were added to the monolayer of Vero cells. After incubation for 1 h at 37°C with 5% CO_2_, the inoculum was removed and replaced by 1 ml of 2.4% CMC in supplemented Earle’s 199 medium. After 7 days of incubation, cells were fixed with 10% formaldehyde, washed, and stained with 0.4% violet crystal for plaque visualization.

### Viral Proliferation Studies

Vero and C6/36 cells were seeded at a density of 40,000 cells/cm^2^ and 80,000 cells/cm^2^ in T-25 flasks, respectively, 24 h before infection. Parental and synthetic viral stocks were diluted in supplemented Earle’s 199 medium for Vero cells or supplemented Leibovitz’s L-15 medium for C6/36 cells. 1 ml of virus inoculum at MOI 0.02 was added to each flask incubated for 1 h at 37°C with 5% CO_2_ for Vero cells, and 28°C for C6/36 cells. After that, the inoculum was removed, and the appropriate culture medium was added to the cell monolayer. Culture supernatant was harvested every 24 h until 5 days post-infection, and the virus was titrated on Vero cells by plaque assay.

### Mouse Infection

AG129 mice (deficient in IFN-α/β and IFN-γ receptors) were obtained from the Institute of Science and Technology in Biomodels (ICTB, Fiocruz). The animals were bred and maintained under specific pathogen-free conditions. The study was carried out in strict accordance with the Guide of the National Council for Control of Animal Experimentation (CONCEA). The protocol was approved by the Committee on the Ethics of Animal Experiments (CEUA) of the Oswaldo Cruz Foundation (Permit Number: L-034/19). Groups of 6- to 8-week-old mixed-sex mice were infected by the ZIKV Rio-U1 isolate and the infectious clone IC.RioU1. Animals were infected in both footpads with 1 × 10^4^ plaque-forming units (PFU) in 60 μl (30 μl/footpad). Mock-infected mice received diluent medium (Earle’s 199 medium supplemented with 25 mM HEPES). The animals were monitored twice daily for 2 weeks. Submandibular blood withdrawals were performed every 2 days to monitor viremia. For both groups, mice were euthanized when severe clinical evidence of disease were observed accordingly to the signs of morbidity described in the Supplementary Table 3. The evaluated symptoms included difficulty in locomotion, hunched stance, ruffled fur, aggressiveness, tremors, dyspnea or tachypnea, and weight loss. The final blood collection was obtained by cardiac puncture while the mouse was under deep anesthesia followed by cervical dislocation. Surviving mice were euthanized 14 days after infection. Average survival time (AST), percent of mortality, clinical scores, and weight variations were calculated.

### Mouse Brain Histopathology

Brains of Mock, Rio-U1, and IC.RioU1 infected animals were collected at the time of necropsy, fixed in Carson’s formalin-Millonig for 48 h (Carson et al., 1973), and processed according to standard paraffin-embedded protocol. Sections of 5 μm were stained with hematoxylin and eosin and examined microscopically under an AxioHome microscope (Carl Zeiss, Germany) equipped with a HRc5 Axiocam digital camera (Carl Zeiss, Germany).

### Viral Load of Animal Samples

For viral load determination, brain specimens and whole blood were collected in RNA (Ambion) and further extracted with RNAqueous-4PCR Total RNA Isolation Kit (Ambion) and QIAamp Viral RNA Mini Kit (Qiagen), respectively. Real-time RT-qPCR was performed using TaqMan Fast Virus 1-Step Master Mix (Applied Biosystems) in an Applied Biosystems StepOnePlus Instrument as previously described (Bonaldo et al., 2016).

### Statistical Analyses

All data were analyzed with GraphPad Prism 8.02 software. An unpaired Student’s *t*-test (two-tailed) was used to determine significant differences in virus titers in growth kinetics, viremia, plaque size, and viral focus area. The differences were only considered significant when *p* < 0.05. For survival analysis, Kaplan–Meier survival curves were analyzed by the log-rank test (Mantel–Cox).

## Results

### Cloning Strategy of Genomic cDNA

In this work, we describe an alternative approach to *in vitro* synthesize ZIKV. The constructed virus was based on the genome sequence of ZIKV strain Rio-U1, which was previously isolated from a urine sample of a pregnant woman (Bonaldo et al., 2016). Initially, four ZIKV cDNA fragments were obtained, covering the full-length genome of the Rio-U1 strain and presenting overlapping regions at either ends (Figure 1). The first synthetic fragment (Z1) was designed to carry the SP6 RNA polymerase promoter directly fused to the viral 5’ UTR (untranslated region) end, followed by the genomic sequences corresponding to the 5′ UTR and most of the structural proteins (ZIKV genome position from 1 to 2,018). The three other synthetic fragments carry the portions of the genome encoding the remaining E protein and all the non-structural proteins that are fragment Z3 (genome position from 1,653 to 4,793), Z4 (from 4,581 to 8,855), and Z2 (from 8,832 to 10,807). Also, the fragment Z2 contains the complete 3′UTR sequence of ZIKV Rio-U1 strain. All cDNA fragments cloned into plasmid vectors (Table 1) include unique restriction endonuclease sites at each terminal cDNA fragments, allowing the direct assembly of genome-length cDNA (Figure 1A).

We utilized the plasmid pCC1-Z3, a low-copy number plasmid, to be a start point for approaching the ZIKV Rio-U1 infectious clone. The stable infectious clone was only possible when developed in a two-plasmid system. Firstly, the plasmid encompassing the central part of the ZIKV genome (pCC1-Z3Z4) was assembled by cloning the Z4 into pCC1-Z3. This construct carries the ZIKV cDNA corresponding to the genome position from 1,653 to 8,855. The second construct brings the fragments Z1 and Z2 representing the 5′ (from 1 to 2,001) and 3′ (from 8,846 to 10,807) ends of the viral genome, respectively. To obtain it, we replaced the fragment Z3 with a polylinker cassette in the pCC1-Z3 plasmid, allowing the cloning of fragments Z1 and Z2, forming the plasmid pCC1-MCS-Z1Z2.

### Genome Assembly and Viral Recovery

The construction of a ZIKV-infectious clone in a genetically stable two-plasmid system was reached. However, since the vector is a single copy plasmid, the mass yield is reduced after DNA extraction. The plasmidial DNA preparations carried bacterial DNA contamination associated with this limitation, hampering the subsequent genome assembly (Supplementary Figure 2). To improve the viral cDNA template production, we associated PCR amplifications with the sequential steps for assembling ZIKV cDNA template. It was initially afforded by PCR amplification of the entire pCC1-Z1Z2 (8,255 bp) and Z3Z4 (7,456 bp) fragments (Figure 1B, Supplementary Table 2, and Supplementary Figure 2). The complete viral cDNA template assembly was performed after cleavage of both amplicons with *Kpn*I and *Mlu*I, followed by DNA ligation. After linearization with *Xho*I and amplification of the synthetic viral cDNA using the primer pair 3, the 3′ cDNA end was generated (Figure 1C and Supplementary Table 2). The forward primer anneals at the SP6 promoter sequence, and the reverse primer anneals at the 3′ UTR end comprising the *Xho*I restriction site and reconstituting the last genomic nucleotide T, which was excised after enzymatic digestion. The size of the template is 10,847 bp containing the ZIKV genomic cDNA of 10,807 bp (Supplementary Table 2).

**FIGURE 2:**
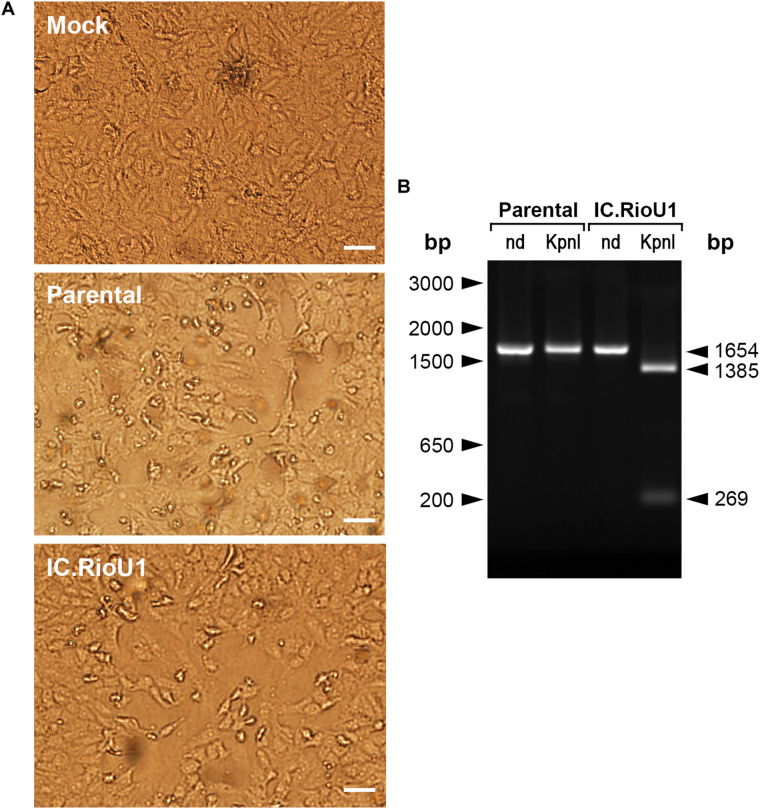
Recovery of synthetic ZIKV after Vero cell transfection. **(A)** Phase-contrast optical microscopy of culture flasks containing mock-infected Vero cells (Mock), positive control of Vero cells infected with ZIKV isolate Rio-U1 (Parental), or electroporated with synthetic ZIKV RNA (IC.RioU1). The white bars represent 100 μm. **(B)** Detection of ZIKV RNA in the extracellular medium by RT-PCR analysis. The electrophoretic profiles of amplicons obtained from (M) mock-infected cells, (P) Vero cells infected with ZIKV isolate Rio-U1, and (IC) synthetic ZIKV RNA. The amplicons were cleaved with *Kpn*I. (nd) which means the control of the amplicons were not submitted to the *Kpn*I digestion. The size marker migration is indicated on the right of the figure.

The full-length ZIKV cDNA was transcribed *in vitro*, and 120 h after transfection in Vero cells, CPE was detected, indicating viral infection. Small round foci and many refractive cells dropping from the monolayer were visualized (Figure 2A). To exclude the possibility that the recovered recombinant ZIKV would consist of a contamination with the parental virus, we treated amplicons (genomic region from 1,732 to 3,385) with the restriction enzyme *Kpn*I. Only IC.RioU1 virus carries the *Kpn*I restriction site, which was one of the single restriction sites created in the development of the ZIKV infectious clone strategy (Figure 1). Digestion of IC.RioU1 amplicon generated two expected fragments of 1,385 bp and 269 bp, while in the parental amplicon we noticed only one band of 1,654 bp (Figure 2B). In conjunction, these results demonstrate that the described methodology is feasible to obtain viable and infectious synthetic ZIKV. Full-genome sequencing of the recovered ZIKV revealed no change other than the expected synonymous mutations at regions that were genetically manipulated in the parental genome of ZIKV Rio-U1.

### Characterization of ZIKV RioU1 Virus Infectious Clone

**FIGURE 3:**
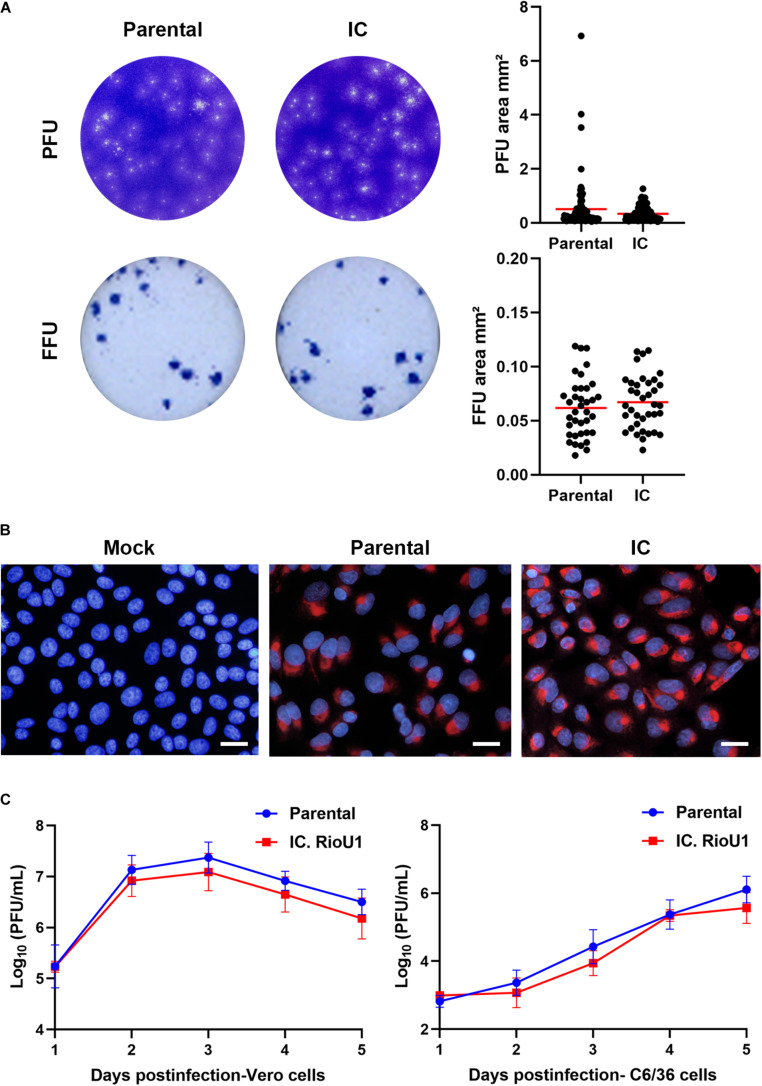
*In vitro* characterization of IC.RioU1 virus. **(A)** Viral plaque and focus morphology of the original isolate Rio-U1 (Parental) and the recovered infectious clone (IC. RioU1). On the right, the scatter plot graphs indicating the individual values for plaque and focus size. Red bars represent the mean of the data sample. **(B)** ZIKV E protein detection after 72 h of Vero cell infection with the parental and IC.RioU1. Infected cells were fixed and processed for immunolabeling with the pan-flavivirus antibody 4G2 (red) and staining cell nuclei with DAPI (blue) White bars indicate 100 μm. **(C)** Comparative studies of replication kinetics of the ZIKV isolate Rio-U1 and the infectious clone counterpart. Mammal Vero cells and mosquito C6/36 cells were infected with the parental and IC.RioU1 ZIKV at MOI of 0.02. Supernatants from infected cells were collected at indicated times post-infection and titers were determined by plaque assay. Each time point represents the mean ± SD of three independent experiments. No significant statistical difference was observed between both viruses (Unpaired *T*-test, Two tailed, *p* > 0.05).

We studied how similar the recovered virus is to its parental virus since the synthetic ZIKV is a genetic clone and could not contain the genetic diversity (quasispecies) expected to be present in ZIKV isolate Rio-U1. We characterized the Vero cell infection pattern of the synthetic ZIKV compared to the parental ZIKV isolate Rio-U1. Firstly, the parental virus and the infectious clone infectivities were determined using standard plaque-forming assay on Vero cells (Figure 3A). Both viruses present a similar plaque size phenotype, the recovered ZIKV displaying a plaque size average of 0.34 ± 0.26 mm^2^ and the parental counterpart of 0.51 ± 0.93 mm^2^. There is no significant difference between the areas of both (*p* = 0.097; Unpaired *t*-Test- Two-Tailed). However, the observed standard deviation around the parental virus plaques is about three and a half times greater than in the clone, suggesting the occurrence of a more significant variation in the plaque size of the parental virus.

In addition, the formation of infectious foci in Vero cells was also compared by immunofocus assay using the pan-flavivirus 4G2 antibody against the envelope protein (Figure 3A). Both viruses have the same infection pattern, with a similar viral focus size average, 0.067 ± 0.024 mm^2^, in the recovered ZIKV and 0.061 ± 0.027 mm^2^ in parental virus (Figure 3A). There is no significant difference between the plaque sizes of both viruses (*p* = 0.383; Unpaired *t*-Test- Two-Tailed). The standard deviation is similar, showing that the viral infection pattern is remarkably similar between viruses.

The observed infection pattern of each virus infection was quite similar when Vero cells were infected and immunostained with the antibody 4G2 at 72 h post-infection (Figure 3B). Both parental and synthetic virus-infected Vero cells displayed the same pattern of perinuclear labeling. Finally, we examined the proliferation capacity in the mammalian Vero cells and the *Aedes albopictus* mosquito C6/36 cells (Figure 3C). The replication profile of the parental virus and its infectious clone was highly similar in both cells. In Vero cells, the viral growth peaks were reached at 72 h post-infection, with the parental virus displaying a titer of 7.37 ± 0.31 log_10_ PFU/ml and the infectious clone, 7.09 ± 0.37 log_10_ PFU/ml, followed by a plateau at 96 h of infection (Figure 3C). There is no significant difference between the viral peaks (*p* = 0.367; Unpaired *t*-Test- Two-Tailed). The proliferation in C6/36 has a different profile, with an ascending curve and peak proliferation in 120 h after infection, with parental virus presenting a titer of 6.11 ± 0.39 log_10_ PFU/mL and the infectious clone, 5.57 ± 0.46 log_10_ PFU/ml. Peak-viral titers in this cell did not show any significant difference (*p* = 0.195; Unpaired *t*-Test- Two-Tailed).

### Virulence in AG129 Mice

**FIGURE 4:**
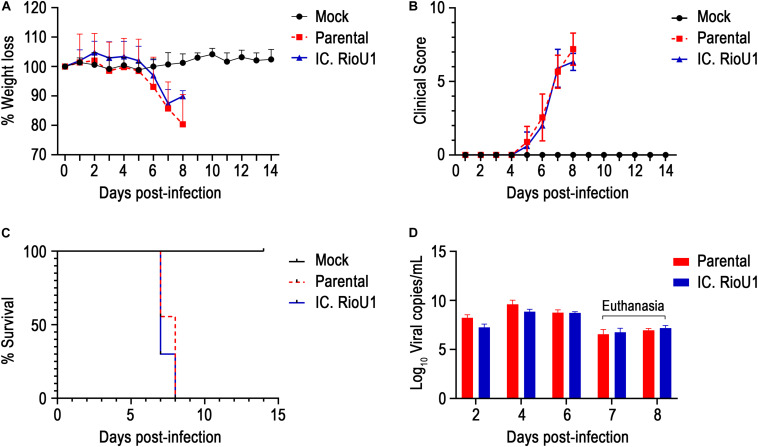
*In vivo* characterization of IC.RioU1 virus using AG129 mouse model. The 6–8-week-old mice were infected with 10,000 PFU in both footpads with the different viruses (Parental and IC.RioU1) or with diluent medium (Mock). Mice were monitored daily for 2 weeks. Morbidity among these animals were measured by **(A)** weight loss and **(B)** clinical scores. **(C)** Deaths were recorded in Kaplan–Meier survival curves. **(D)** Viremia was evaluated at days 2, 4, and 6 post-infection and at the moment of death. At the seventh day, four mice inoculated with parental virus and seven with IC.RioU1 virus were euthanized. At the eighth day, five mice inoculated with parental virus and three with IC.RioU1 virus reached a humane endpoint. Clinical signs were scored as described in the Section “Materials and Methods,” and Supplementary Table 3. Error bars represent SD of the mean for each group of mice. Differences in morbidity and viremia of the two viruses were not statistically significant (Unpaired *t*-test – Two tailed, *p* > 0.05), the same was observed in survival curve (Long-rank test).

We compared the virulence of the parental and recombinant ZIKV in the AG129 mouse model. Subcutaneous infection with both viruses (10^4^ PFU) led to weight loss at 6 days post-infection (dpi) (Figure 4A). Both groups exhibited a progressive increase in clinical score from day 5 (Figure 4B), with disease characterized by difficulty in locomotion, a hunched stance, aggressiveness, tremors, dyspnea/tachypnea, and weight loss. At 7 dpi, 100% mortality occurred in both groups (Parental: 7.6 ± 0.5 days and IC.RioU1 7.3 ± 0.5 days) (Figure 4C). There was no statistical difference in the average survival time (AST) between mice inoculated with both viruses (*p* = 0.273, Log-rank test). Weight loss data were consistent with survival data. As expected, uninfected controls did not exhibit weight loss and clinical signs of disease and survived until the end of the experimentation time.

**FIGURE 5:**
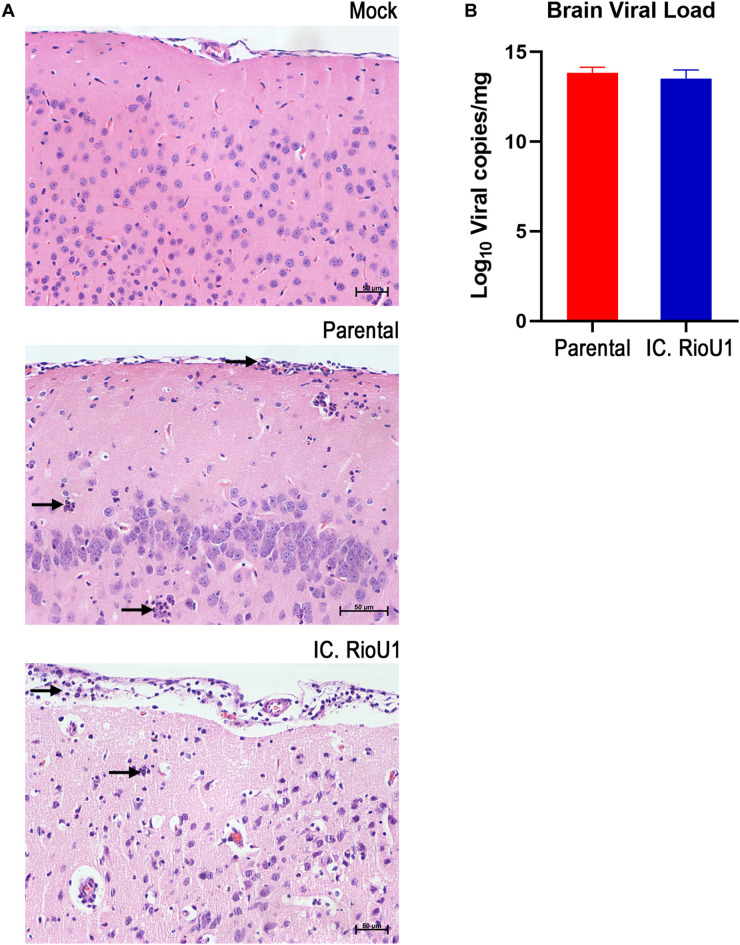
Neurotropism of the ZIKV Rio-U1 (Parental) and the Infectious cDNA Clone (IC.RioU1) in AG129 mice. **(A)** Brain histopathological analysis after mock, ZIKV Rio-U1 (Parental), and IC.RioU1 infection. The brains of the Parental and IC.RioU1 infected animals exhibited inflammatory infiltrate in the cortex and meninges. Neutrophilic infiltration is indicated by black arrows. Data are representative of two independent experiments (*n* = 4 and 5). The brain sections were stained with hematoxylin and eosin. Scale bar, 50 μm. **(B)** Viral load observed in the brains, of both groups, at time of death.

To further explore ZIKV-induced disease, we determined viremia at days 2, 4, and 6 post-infection and at the time of death (euthanasia). The animals were euthanized when their clinical scores reached 6 as described in Supplementary Table 3. Both mouse groups inoculated with the parental and infectious clone viruses displayed a critical endpoint at days 7 (Parental, *n* = 4 deaths; IC.RioU1, *n* = 7 deaths) and 8 (Parental, *n* = 5 deaths; IC.RioU1, *n* = 3 deaths). As observed in Figure 4D, viral load ranged from 6.8 log_10_ to 9.8 log_10_ viral RNA copies/ml reaching the peak of viremia at day 4 for both viruses, with no significant difference in viral titers (*p* = 0.398, Unpaired *t*-Test- Two-Tailed). Since in the AG129 mouse model of ZIKV infection, the main target organ is the brain, we measured the viral load in this tissue on the day of death, as shown in Figure 5B. The viral load of animals infected with the parental or infectious clone is exceptionally high, reaching a load of 14 log_10_ viral copies/mg with no significant difference between the viruses (*p* = 0.253, Unpaired *t*-Test- Two-Tailed).

**FIGURE 6:**
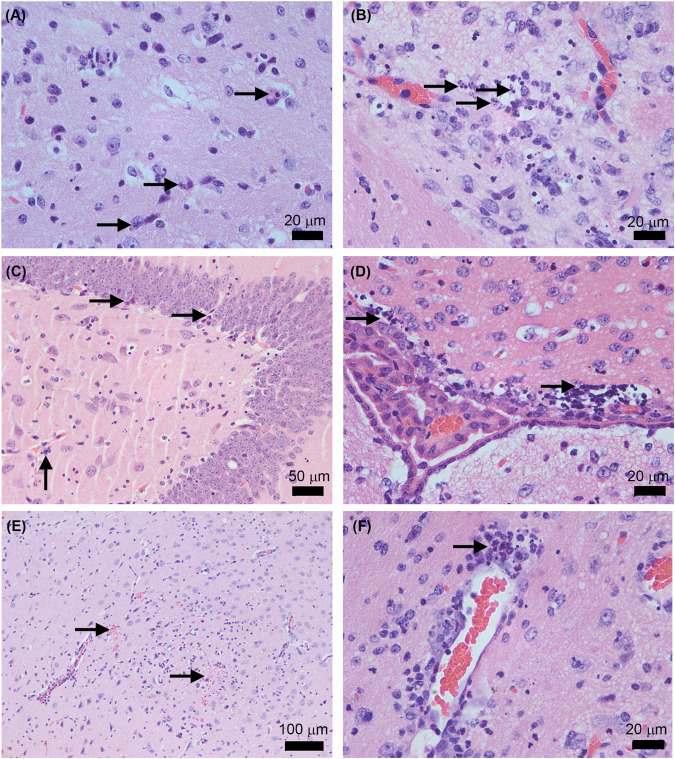
Histopathological features in AG129 mouse brain after the infection with the ZIKV Rio-U1 (Parental) and the Infectious cDNA Clone (IC.RioU1). Karyorrhexis in the cortex of IC.RioU1 **(A)** and Parental **(B)** infected animal. Nuclear pyknosis and karyorrhexis in the hippocampus **(C)** and bordering the choroid plexus **(D)**. Brain hemorrhage foci **(E)**. Perivascular cuffs with a neutrophilic predominance **(F)**. The images are representative of IC.RioU1 **(A,C)** and Rio-U1 **(B,D–F)** infection. Black arrows indicate the histopathological features observed in each panel **(A–F)**. The brain sections were stained with hematoxylin and eosin. Data are representative of two independent experiments.

To better characterize the lesions provoked by the ZIKV acute infection in the brain, we proceeded with a histopathological analysis of animals infected with the parental and infectious clone. Under microscopy analysis, both viruses led to the same histopathological findings. Brains of all infected animals reveled meningoencephalitis (Figure 5A), exhibiting neutrophilic inflammatory infiltrate. Pycnotic and karyorrhexis nuclei were observed in extensive areas of the cortex (Figures 6A,B), hippocampus (Figure 6C) and also bordering the choroid plexus (Figure 6D). Small hemorrhagic foci (Figure 6E) and the formation of perivascular cuffs with the presence of neutrophils were also observed (Figure 6F). Both viruses led to the same histopathology, and the lesions caused by ZIKV replication in the brain are compatible with the mouse neurological signs observed during disease.

## Discussion

In 2016, Latin America and the Caribbean experienced an unprecedented Zika epidemic. The ZIKV emerged as a main human pathogen due to its association with the impairment of perinatal development and Guillain–Barré syndrome. The occurrence of these severe cases of Zika points to the significance of studies for understanding the molecular determinants of flavivirus pathogenesis. Therefore, the establishment of molecular tools and experimental models have been developed by many groups to study aspects related to reemergence, replication, and transmission of this virus. Here, we report an infectious clone construction through reverse genetics based on the ZIKV Rio-U1 isolated from the urine of a pregnant woman during the ZIKV epidemic in Rio de Janeiro, Brazil, in 2016, who lived in a region with a high incidence of congenital Zika syndrome (Bonaldo et al., 2016). Previous studies support the importance of investigating the ZIKV Rio-U1 through infectious clone technology (Fernandes et al., 2016; Campos et al., 2017; Magnani et al., 2018; Messias et al., 2019, 2020; Panganiban et al., 2020). ZIKV Rio-U1 displayed high infection and dissemination rates in *Aedes aegypti*, leading to the very high viral transmission after day 14 of virus exposure (Fernandes et al., 2016). Besides, orally-infected mosquito females by Rio-U1 strain can also venereally transmit the virus to males (Campos et al., 2017). In another study, the ZIKV Rio-U1 virus caused lethality in AG129 mice at lower doses than other common American ZIKV isolates (Magnani et al., 2018). The infection of pregnant macaques with this isolate resulted in maternal viremia, the virus crossing into the amniotic fluid, and in uterus fetal deaths (Magnani et al., 2018).

Reverse genetics has been extensively used to answer several questions regarding the consequences of ZIKV infection in developmental biology. However, unlike other single-stranded RNA viruses, this tool’s development for the members of the *Flavivirus* genus has been a significant problem (Tsetsarkin et al., 2016). It has been reported that structural protein E and NS1 regions of flavivirus cDNA may contain *E. coli* promoters and produce proteins that cause toxicity to the recipient bacteria (Pu et al., 2011). Several approaches have been taken to reduce this toxicity, including (i) inactivation of cryptic promoters for bacterial RNA polymerase in the flavivirus genome using silent mutagenesis (Münster et al., 2018; Zhao et al., 2018; Pallares et al., 2020); (ii) insertion of intron sequences to disrupt the viral ORF (Schwarz et al., 2016; Tsetsarkin et al., 2016); (iii) *in vitro* binding (Widman et al., 2017; Gorman et al., 2018) or Gibson assembly that allows cloning genome fragments with later assembly (Setoh et al., 2017; Weger-Lucarelli et al., 2017a, b); and finally (iv) cloning a full-length cDNA into a low-copy vector (Shan et al., 2016; Annamalai et al., 2017; Mutso et al., 2017; Shan et al., 2017b), such as the use of artificial bacterial chromosome plasmids (Mutso et al., 2017; Márquez-Jurado et al., 2018). More recently, the ISA method (Infectious Subgenomic Amplicons) was developed (Aubry et al., 2014), which is performed with transfection of fragments of double-stranded cDNA that covers the entire genome of the RNA virus, bypassing cloning steps in bacteria (Atieh et al., 2016; Gadea et al., 2016). Unfortunately, this method could be associated with low efficiency of recombination of the cDNA fragments into transfected cells. Therefore, the amount of virus produced after the transfection using the ISA approach could be low (Ávila-Pérez et al., 2018).

Initially, we attempted to build the infectious clone based on the strategy developed previously in our laboratory for the vaccine yellow fever 17D virus. The genome was cloned in derivatives of either pBR322 or pACN118 plasmids (Rice et al., 1989; Bonaldo et al., 2002, 2005). The ZIKV cDNA was split into two fragments, one containing the central part of the genomic cDNA and the other the 5′ and 3′ regions. However, cloning the ZIKV genome in this format was not feasible because they were genetically unstable. Therefore, following the same rationale, the genomic cDNA was assembled into two plasmids but employing a low copy plasmid, a derivative of pCC1-Fos. The major constraint in working with this kind of vector is the low amount of viral cDNA, thus influencing the yield of viral cDNA template and, subsequently, the recovery of viral particles after transfection (Shan et al., 2016). To overcome this hurdle, we associated PCR-amplifications in different viral cDNA generation steps to increase the genomic cDNA mass. The intermediate plasmids pCC1-MCS-Z1Z2 and pCC1-Z3Z4 were amplified by PCR with highly processing and proofreading enzymes. After the complete template assembly, followed by further PCR amplification, it was possible to generate large amounts of cDNA and subsequent viral RNA. The amplification steps facilitated the viral recovery after 5 days of Vero cell transfection, totally in agreement with what has been described elsewhere (Shan et al., 2016; Mutso et al., 2017). The clone-derived virus presented no genetic alterations compared to the genome of the parental virus, except for those corresponding to the restriction sites used in the cloning steps. This methodology allowed the generation of cDNA-derived ZIKV with remarkably close genetic and biological properties to the parental strain from which it was constructed. The synthetic virus displayed quite similar infection patterns in mammalian and insect cells. We did not observe any significant differences either in viral proliferation profiles or E protein-labeling patterns, according to other studies describing alternative strategies to obtain ZIKV infectious clones (Schwarz et al., 2016; Annamalai et al., 2017; Deng et al., 2017; Liu et al., 2017; Mutso et al., 2017; Weger-Lucarelli et al., 2017b; Chen et al., 2018). However, some studies showed that cDNA-derived Zika viruses exhibited more attenuated replication kinetics than parental viruses (Shan et al., 2016; Tsetsarkin et al., 2016; Widman et al., 2017). It might be due to the limited genetic heterogeneity of the recombinant virus population and the more genetically diverse quasispecies of the parental virus (Shan et al., 2016). In the present work, these features were evaluated by plaque phenotype assays. We observed that the parental virus displayed a more heterogeneous plaque morphology than the ZIKV-infectious clone. This difference in plaque sizes was not surprising because the recombinant virus was derived from a homogenous RNA transcript population, whereas the parental virus is presumably also composed of quasispecies. This result follows what was previously described (Shan et al., 2016).

We assessed the infectivity and virulence of the infectious clone in comparison with the parental ZIKV Rio-U1 in the AG129 mouse model. Several immunocompromised adult mouse models have been reported to support the replication of ZIKV; among those, AG129 showed greater susceptibility and more severe disease upon ZIKV infection (Aliota et al., 2016; Rossi and Vasilakis, 2016). We observed practically identical mortalities, with similar disease progression and morbidity in the animals infected with the parental and the cDNA-derived virus. Also, viremia over the days was similar, but with a slight decay in mice infected with the ZIKV clone, reflecting the cell models’ results without any statistical significance. Our results are in line with Weger-Lucarelli et al. (2017a), who compared the PRVABC59 ZIKV isolate with its infectious clone in model AG129 (deficient in IFN-α/β and IFN-γ receptors) and observed similar pathogenesis between them. However, in some cases, mouse infection with the cDNA-derived viruses appears to be less severe, exhibiting a slower mortality curve and later clinical signs of disease than the respective viral isolate (Shan et al., 2016; Widman et al., 2017; Ávila-Pérez et al., 2018). It could be due to the limited genetic heterogeneity of the recombinant virus population and the more genetically diverse quasispecies composition of the parental virus, a known virulence determinant (Vignuzzi et al., 2006).

ZIKV targets proliferative cells in neurogenesis sites, causing injury, as previously determined in studies with immunocompromised mice (Shaily and Upadhya, 2019). When myelinating cultures from Ifnar1 KO mice, all central nervous system cells were vulnerable to ZIKV infection, especially oligodendrocytes (Cumberworth et al., 2017). On the other hand, the infection of non-neuronal cells, like primary human astrocytes and microglia, resulted in high viral replication and the induction of elevated levels of proinflammatory immune cytokines. It may contribute to the neurodevelopmental impact on the brain (Hamel et al., 2017; Lum et al., 2017). In the present work, we demonstrated that both viruses were highly replicative in AG129 mouse brain, as observed in Dowall et al. (2016), leading to a rapid disease progression. It is described that ZIKV infection in the AG129 mice leads to findings that display similarities with the disease in humans, such as the development of meningoencephalitis, fetal brain malformations (Miner and Diamond, 2017), neuronal apoptosis (Dowall et al., 2016), and scattered neutrophils in the nervous system, with perivascular infiltration and focus adjacent to the choroid plexus (Aliota et al., 2016). Our study is in line with all these described findings. Therefore, we can conclude that both the parental virus and its clone led to similar pathogenesis in an animal model, presenting characteristic findings of ZIKV infection.

Here, we developed a genetically stable and efficient infectious clone of ZIKV based on the ZIKV Rio-U1 isolated in the 2016 epidemic in Brazil. This clone could be a tool for genetic and functional studies *in vitro* and *in vivo* to better understand viral infection and pathogenesis. The tools used in the development of this infectious clone may inspire the construction of reverse genetics systems for other flaviviruses.

## Data Availability Statement

The datasets generated for this study can be found in online repositories. The names of the repository/repositories and accession number(s) can be found below: https://www.ncbi.nlm.nih.gov/genbank/, KU926309.

## Ethics Statement

The animal study was reviewed and approved by Ethics Committee of Animal Use at Instituto Oswaldo Cruz (CEUA license L034/2019).

## Author Contributions

IM, DF, and MB: conceive the study. IM, DF, NF, AS, MS, and MB: establishment of the cloning strategy. IR: real -time PCR and analysis. LR, MN, SC, and AR: technical support of the study. PM and MP-M: histopathological analysis and board preparation. IM: statistical analysis. IM, DF, NF, and MB: prepared figures, tables, and/or Supplementary Material. IM, DF, and MB: prepared the manuscript. All authors critically read and approved the final version of the manuscript.

## Conflict of Interest

The authors declare that the research was conducted in the absence of any commercial or financial relationships that could be construed as a potential conflict of interest.
