# Supplementary material for: Recovery of Synthetic Zika Virus Based on Rio-U1 Isolate Using a Genetically Stable Two Plasmid System and cDNA Amplification
Source: Front Microbiol. 2021 Feb 24;12:639655. doi: 10.3389/fmicb.2021.639655 (PMC7943741; doi:10.3389/fmicb.2021.639655)
Supplement: Supplementary file 1 [file Data_Sheet_1.pdf]

Supplementary information of “**Recovery of synthetic Zika virus based on Rio-U1 isolate using a genetically stable two plasmid system and cDNA amplification**”

Iasmim Silva de Mello1&, Déberli Ruiz Fernandes1&, Nathália Dias Furtado1, Alexandre Araújo Cunha dos Santos1, Marta Pereira dos Santos1, Ieda Pereira Ribeiro1, Lidiane Menezes Souza Raphael1, Mônica da Silva Nogueira 2, Stephanie Oliveira Diaz da Cruz1, Adalgiza da Silva Rocha3, Pedro Paulo de Abreu Manso 4, Marcelo Pelajo Machado4 and Myrna Cristina Bonaldo1*

1Laboratório de Biologia Molecular de Flavivírus, Instituto Oswaldo Cruz, Fundação Oswaldo Cruz, Rio de Janeiro, Brazil

2Centro de Experimentação Animal, Instituto Oswaldo Cruz – FIOCRUZ

3Central Analítica, Unidade de Apoio ao Diagnóstico do COVID-19 - UNADIG-RJ, Vice-Presidência em Produção e Inovação - FIOCRUZ

4Laboratório de Patologia, Instituto Oswaldo Cruz, Fundação Oswaldo Cruz, Rio de Janeiro, Brazil

*** Correspondence:**

Myrna Cristina Bonaldo

mbonaldo@ioc.fiocruz.br

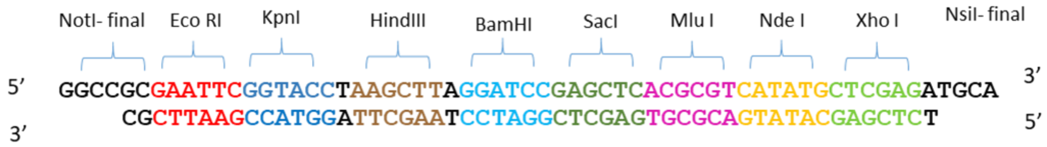

Supplemental Figure 1 – Pair primers utilized to construct pCC1-MCS-Z1Z2 containing a polylinker sequence. The original low-copy plasmid pCC1-Z3 was digested with the restriction enzymes NotI and NsiI, and the Z3 fragment was replaced with the polylinker with cohesive ends NotI and NsiI. Using the new plasmid named pCC1-MCS, we subsequently cloned the ZIKV cDNA Z1 and Z2 with NotI and KpnI and MluI-XhoI cohesive ends, respectively.

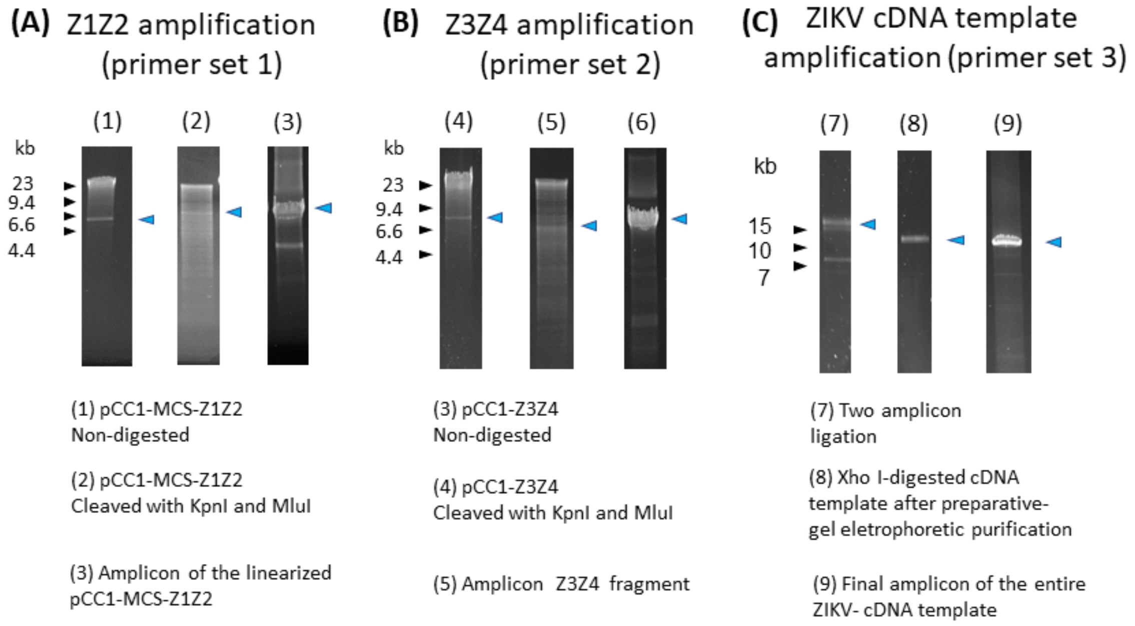

Supplemental Figure 2 – ZIKV infectious clone construction. The construction of a ZIKV-infectious clone was reached by developing a genetically stable two-plasmid system. The plasmidial mass yields after DNA extraction of pCC1-MCS-Z1Z2 (lane A1; 8,255 bp; DNA mass 17.2 ng/ul) and Z3Z4 (lane B1; 7,456 bp; DNA mass 26.8 ng/ul) were reduced, hampering the subsequent genome assembly. The quantifications were overestimated, considering the inevitable contamination with chromosomal DNA. The cleavage with *Kpn*I and *Mlu*I of the plasmid preparations, necessary to construct the complete ZIKV infectious clone, produced barely detected bands (indicated by blue arrows) of the digested plasmids (lane A2 and lane B5). To circumvent this constraint, we directly PCR amplified the pCC1-MCS-Z1Z2 and Z3Z4 fragments as indicated by blue arrows in lanes A3 (27.8ng/ul) and B6 (8.52 ng/ul), respectively. After the digestion of the amplicons with *Kpn*I and *Mlu*I, the fragments were ligated (lane C7, blue arrow). In a further step, we cleavaged the ligated DNA with *Xho*I and purified the template band by preparative gel electrophoresis (lane C8, blue arrow; DNA mass 1,99 ng/ul). We proceeded with the PCR amplification of the synthetic viral cDNA using primer pair 3, generating the complete template of 10,847 bp containing the ZIKV genomic cDNA of 10,807 bp (lane C9, blue arrow, DNA mass 82.2ng/ul).
