# Supplementary material for: Recovery of Synthetic Zika Virus Based on Rio-U1 Isolate Using a Genetically Stable Two Plasmid System and cDNA Amplification
Source: Front Microbiol. 2021 Feb 24;12:639655. doi: 10.3389/fmicb.2021.639655 (PMC7943741; doi:10.3389/fmicb.2021.639655)
Supplement: Supplementary file 2 [file Data_Sheet_2.pdf]

Supplementary information of "**Recovery of synthetic Zika virus based on Rio-U1 isolate using a genetically stable two plasmid system and cDNA amplification**"

Iasmim Silva de Mello1&, Déberli Ruiz Fernandes1&, Nathália Dias Furtado1, Alexandre Araújo Cunha dos Santos1, Marta Pereira dos Santos1, Ieda Pereira Ribeiro1, Lidiane Menezes Souza Raphael1, Mônica da Silva Nogueira 2, Stephanie Oliveira Diaz da Cruz1, Adalgiza da Silva Rocha3, Pedro Paulo de Abreu Manso 4, Marcelo Pelajo Machado4 and Myrna Cristina Bonaldo1*

1Laboratório de Biologia Molecular de Flavivírus, Instituto Oswaldo Cruz, Fundação Oswaldo Cruz, Rio de Janeiro, Brazil

2Centro de Experimentação Animal, Instituto Oswaldo Cruz – FIOCRUZ

3Central Analítica, Unidade de Apoio ao Diagnóstico do COVID-19 - UNADIG-RJ, Vice-Presidência em Produção e Inovação - FIOCRUZ

4Laboratório de Patologia, Instituto Oswaldo Cruz, Fundação Oswaldo Cruz, Rio de Janeiro, Brazil

*** Correspondence:**

Myrna Cristina Bonaldo

mbonaldo@ioc.fiocruz.br

Supplemental Table 1. Set of primers employed in part or the entire ZIKV cDNA template amplification.

Target	Primer	Sequence 5' → 3'	Amplicon (bp)
pCC1-MCS-Z1Z2	1F	CCTAAGCTTAGGTACCTTGCAAGGTCCATC	8255
	1R	GCTTAGGATCCGAGCTCACGCGTCTGTACC	
pCC1-Z3Z4	2F	GGATGTGCTGCAAGGCGATTAAGTTGG	7456
	2R	CTCGTATGTTGTGTGGAATTGTGAGC	
IC.Rio-U1	3F	CCTTATGTATCATACACATACGATTTAGGTGACACTATAGAG	10847
	3R	AGACCCATGGATTTCCCCACACCGG	

Supplemental Table 2 – Primer set designed for RT-PCR and viral genome sequencing.

Primer	Position	Sequence 5' → 3'
ZK1F	1 - 25	AGTTGTTGATCTGTGTGAATCAGAC
ZKGAP5'AR	166 - 182	GCTCACACGGGCTACTC
ZK1AR	623 – 644	ATCCAGCATAGGGCATTCATAG
ZK1AF	627 – 646	GAATGCCCTATGCTGGATGA
ZK1BF	982 – 1003	GGTGCATAGGAGTCAGCAATAG
ZK1BR	1007 – 1028	ACCTGACATACCTTCCACAAAG
ZK1CR	1202 – 1221	GGTAGGCTTCACCTTGTGTT
ZK1CF	1373 – 1395	AGAGAATCTGGAGTACCGGATAA
ZK2F	1732 – 1751	GGCAAACTGTCGTGGTTCTA
ZK1R	1872 – 1892	GTATGACACGCCCTTCAATCT
ZK2AF	2431 – 2450	CCCTTATGTGCTTGGCCTTA
ZK2AR	2434 – 2453	CCCTAAGGCCAAGCACATAA
ZK24L1F	2608 – 2626	GTAGATTGGCAGCAGCAGT
ZK2BR	2731 – 2751	CCGTCAGTTGAACTCCATTCT
ZK24L1R	2927 – 2948	AAAGCTGTTCCATGCTCTATGT
ZK3F	3085 – 3107	GCTACTGGATTGAGAGTGAGAAG
ZK2R	3364 – 3385	CTCAGAGATGGTCCTCTTGTTC
ZK3AR	3743 – 3764	ATCTCCTCCAGTGTTCATTTCC
ZK24L2F	3807 – 3827	GCGTTGCTGGTATCTTTCATC
ZK3AF	3972 – 3992	GTTGTTCCACGCACTGATAAC
ZK24L2R	4045 – 4061	TGCTCTCCACGCCACAA
ZK4F	4345 – 4366	GTTACGTGGTCTCAGGAAAGAG
ZK3R	4548 – 4567	GGGTATGGCTATTGGGTTCAT
ZK4BF	4715 – 4735	ACAAGTTGGAGTGGGAGTTAT
ZK4AR	5026 – 5047	CTCCCACACTTGTCTAGGATTG
ZK4AF	5034 – 5055	GACAAGTGTGGGAGAGTGATAG

ZK4BR	5358 – 5377	GTTCCAGAGTGGGTGACATT
ZK5F	5593 – 5613	CGGACTCCAACTCACCAATTA
ZK4R	5912 – 5933	GACTCTCTCGCCATCAAGTATG
ZK5AR	6277 – 6299	GCACCATCTTCTATCTGTGTAGG
ZK5AF	6282 – 6304	ACAGATAGAAGATGGTGCTTTGA
ZK5BR	6518 – 6537	TGGCTTCCTGGAATCTCTCT
ZK6F	6959 – 6980	TCTAATGGGAAGGAGAGAGGAG
ZK5R	7104 – 7125	CCATCGCCATTAAGGAGTAGTT
ZK6AF	7607 – 7628	CTTGGCTGGAGCTTCTCTAATC
ZK6AR	7607 – 7628	GATTAGAGAAGCTCCAGCCAAG
ZK6BF	8018 - 8039	AAGCTATGGGTGGAACATAGTC
ZK6BR	8062 - 8081	CTCAGCCGCCATATGAAAGA
ZK7F	8549 – 8569	CTTTGACGAGAACCACCCATA
ZK6R	8689 – 8708	GGTGTCGGTCATGGCTATTC
ZK7AF	9187 – 9206	GTGTTGAAGGGCTGGGATTA
ZK7AR	9228 – 9249	CTGGTATGCGACTCATCTCTTC
ZK7BF	9514 – 9534	ACCTAGTGGTGCAACTCATTC
ZK7BR	9537 – 9558	GAACTTCCTCAGCCTCCATATT
ZK8F	10034 – 10054	GGTTCCAACTGGGAGAACTAC
ZK7R	10205 – 10227	CTATGAGAGATCCACACCACAAG
ZKGAP3’F	10643 – 10664	TGGATCTCCAGAAGAGGGACTA
ZK8R	10788 – 10807	AGACCCATGGATTTCCCCAC
zkmelt65f	10034 - 10068	GGTTCCAACTGGGAGAACTACCTGGTCAATCCATG
zkmelt65r	10782 - 10807	AGACCCATGGATTTCCCCACACCGGC
zkmelt70f	10034 - 10090	GGTTCCAACTGGGAGAACTACCTGGTCAATCCATG GAAAGGGAGAATGGATGACCAC
zkmelt70r	10778-10807	AGACCCATGGATTTCCCCACACCGGCCGCC

Supplemental Table 3 - Clinical scores for monitoring signs of disease in AG129 mice

**Observation**	**Score**
**Body weight**	
Loss of 5-10%	1
Loss of 10-15%	2
Loss of 16-20%	3
Loss of 20% or more	6
**Fur**	
Slightly ruffled	2
Evidently ruffled	3
**Respiration**	
Accelerated breathing (tachypnea)	1
Difficult breathing (dyspnea)	3
**Clinical complications**	
Tension during handling	1
Tremors, breath noises, aggression, vocalizations	3
**Motility**	
Abnormal walking and posture (slightly hunched, reduced activity)	1
Massively abnormal walking and posture (evidently hunched, moderately reduced activity)	2
Motility only after stimulation, isolation, lethargy	3
No activity for more than 24 hours	6

**Measures according to total score**	
**3**	Animals are carefully observed, with monitoring 2 times a day
**6**	Implementation of humane endpoint, abort experiment (euthanasia)
